# Walk-In Appointments: An Observational, Cross-Sectional Study of Family Medicine in Central Portugal

**DOI:** 10.7759/cureus.86718

**Published:** 2025-06-25

**Authors:** João L Guedes, Rebeca Cunha, Luiz Miguel Santiago

**Affiliations:** 1 Family Medicine, Unidade de Saúde Familiar (USF) Santo André de Poiares, Vila Nova de Poiares, PRT; 2 Family Medicine, Unidade de Saúde Familiar (USF) Trilhos Dueça, Miranda do Corvo, PRT; 3 Family Medicine, Centro de Estudos e Investigação em Saúde, Universidade de Coimbra, Coimbra, PRT

**Keywords:** acute disease appointment, family health unit, intersubstitution appointment, primary health care, walk-in appointment

## Abstract

Introduction: Walk-in appointments (WIA) are intended to respond to acute situations or exacerbations of chronic pathology and are part of the clinical activity of a family doctor. It is necessary to know the reasons that lead patients to resort to this type of consultation and to understand whether these reasons are suitable for it, in accordance with the internal regulations of the two family health units (FHUs), so that we can properly adjust the offer of this kind of appointment or correct mistakes if it isn't being properly used.

Objectives: Our goal was to characterize WIA in two FHUs during the first half of 2022, according to the frequency of reasons for the consultation, diagnosis using ICPC-2, patient referral, and adequacy of this type of appointment in accordance with the internal regulations of each FHU.

Methods: An observational cross-sectional study was performed, whose population was the patients observed in WIA from January to June 2022 in each FHU. Data was collected by a resident doctor in each FHU. An analysis was performed, anonymously, in accordance with the approved protocol. Descriptive and inferential statistics were performed.

Results: In a sample of 363 WIA, most patients were women (219 (60.3%)), and the mean age was 47.5 years. The highest number of appointments took place on Tuesdays (80 (22.0%)) and in May (73 (20.1%)). The most frequent reason for consultation was R05 “Cough” (50 (13.8%)), and the most frequently coded assessment was A98 “Health maintenance/prevention” (76 (20.9%)). Of the studied appointments, 144 (39.7%) did not meet the established criteria for this kind of appointment.

Conclusion: There is a need to invest in health education, so that the public can be made aware of the specific indications for WIA versus a scheduled appointment. It is also crucial to emphasize the importance of complete, clear, and standardized consult notes associated with accurately classifying using ICPC-2.

## Introduction

Primary care is defined as the population's first level of contact with the health system [1]. It is characterized by addressing the community's main health problems, providing preventive, curative, and rehabilitation services [1].

Family Medicine (FM) is a person-centered specialty, oriented towards the individual, the family, and the community, with Primary Health Care (PHC) as its area of work [2]. It deals with all health problems, in their physical, psychological, social, and cultural aspects, regardless of the person's age, gender, or other characteristics [2]. It manages both acute and chronic problems and is the interface with other specialties, whenever necessary [2].

The role of a Family Doctor (FD) is therefore health promotion, disease prevention, and the provision of curative care and follow-up or palliation, directly or indirectly through the services of others, depending on health needs [2].

In the doctor-patient relationship, there is an encounter between two different perspectives on the concept of illness, which must be discussed and negotiated at each consultation [3,4]. The patient's concept of illness corresponds to what “the patient feels when they go to the doctor”, while the doctor's concept of illness is used to classify “what the patient has when they go home after their appointment with the doctor” [3,4]. The concept of pain should also be taken into account, i.e., the psychosocial effects of the illness, the pain and suffering that the illness causes the patient [3]. According to some authors, in a person-centered consultation, four dimensions of pain should be assessed: the fears and feelings related to the problem, the explanations for the complaints, the impact of the problem on daily life, and the patient's expectations of the doctor [3,5].

In the family health unit (FHU) model of work, patients have various types of appointments available to them. These can be scheduled (FM, risk group, and vulnerable group consultations) or unscheduled (walk-in appointments, WIA). The latter should be activated when there is a need to respond to an acute situation or the exacerbation of a chronic pathology, and are scheduled on a first-come, first-served basis, in consultations held on the same day at specific times by the respective FD and are called “acute disease appointments” (ADA), and are the initiative of the patient. These consultations can also be carried out by another doctor from the FHU team on an inter-substitution basis (inter-substitution appointments, ISA), when there are no more vacancies for the Family Doctor that day, and it may happen that ISA are carried out by the FD himself when the vacancies for ADA are exhausted [6,7].

In Portugal, there were two studies carried out in 2002, which tried to characterize the use of PHC services by studying “urgent consultations” [8,9].

In 2018, another study evaluated the profile of the user of WIA, as well as the main reasons for attending them, and a more recent one evaluated and characterized WIA according to the frequency of reasons for consultation/diagnosis and the destination of users [6,7]. All of them found that women were the main users of WIA, with respiratory infections being the main diagnosis for recurrence to WIA [6-9].

In the 2018 study, the main reason for going to the WIA was “Administrative Procedure” (ICPC-2, A62), thus demonstrating the high number of patients going to the WIA for reasons not directly related to the acute illness, followed by “Cough” (ICPC-2, R05) [7]. In addition, the patients who went to the WIA the most were professionally “active” and January was the month with the highest percentage of this type of consultation [7]. In the 2021 study, it was found that when such a consultation was carried out by the respective FD, there was less referral to a hospital consultation than when the consultation was carried out by another doctor at the FHU and that the main reason for referral was “Shortness of breath/dyspnea” (ICPC-2, R02) [6]. In addition, the frequency of WIA was characterized according to the month, day of the week and time period, with a higher turnout in January on Mondays and in the early hours of the day [6].

None of the studies analyzed the appropriateness of the reasons for scheduling the WIA, and the population context of these studies was large urban centers [6,7].

From our clinical experience, there was an idea that many of these WIA should not have been scheduled as such, and so we want to test and verify this. So, we asked the question: "Are WIA being properly used?"

The aim of this study, carried out in two FHUs in the context of country town, in the ACeS (Grouping of health centres) Pinhal Interior Norte, was to evaluate and characterize, for the first six months of 2022, the profile of WIA users, the reasons for such a consultation, the diagnosis made, the destination of users after the WIA and, according to the internal regulations of each FHU, whether the consultation had been properly used.

## Materials and methods

An observational, cross-sectional study was carried out on a representative, random, and replacement sample of ISA and ADA in the first six months of 2022, at the Santo André de Poiares FHU (SAPFHU) and Trilhos Dueça FHU (TDFHU), of the ACeS (Grouping of health centers) Pinhal Interior Norte.

The universe was all ADA or ISA consultations at the FHUs. The sample size was calculated, with a 95% confidence level and a 5% margin of error.

Exclusion criteria were omission of data or errors detected in the recorded data. In this case, the immediately previous or subsequent case was analyzed if the previous one also had exclusion criteria. If both had exclusion criteria, the initial randomization was maintained.

The data was collected anonymously by specialty residents capable of such research. Data on the WIA was collected from the SClínico® and MIM@UF® computer platforms. The following data was obtained: age and gender of those attending, the time distribution of the WIA in terms of month, day of the week and time slot, the identification of the doctor who carried out the consultation, the type of consultation (ADA or ISA), the reason for the consultation according to the ICPC-2 classification, either expressed or transformed into an ICPC-2 according to the notes in S (from SOAP (Subjective, Objective, Assessment and Plan) notes), and the suitability of the type of consultation according to the FHU regulations. The ICPC-2 diagnosis issued by the ADA or ISA was also obtained, as well as the referral of the patients according to what was described in P of the records. The data on users registered in the units was collected from the MIM@UF® platform in terms of gender, age, and professional status.

As this was a consultation of secondary databases, no terms of free and informed consent were applied, and a favorable opinion was obtained from the Ethics Committee of the Centro Regional Health Administration, as well as authorization from the FHU Coordinators.

The data was analyzed using IBM SPSS Statistics for Windows, Version 27 (Released 2020; IBM Corp., Armonk, New York, United States), using descriptive and inferential statistics, the latter non-parametric for ordinal variables and numerical variables with a non-normal distribution using the Mann-Whitney U test and Fisher's exact test for nominal variables.

A random code was created for coding users who used the WIA, and the database is password-protected and will be kept active for the legal period.

## Results

In the period between January 1st and June 30th, 2022, 17,260 face-to-face consultations were held, 9,395 at TDFHU and 7,865 at SAPFHU, of which 6,464 (37.45%) were WIA (4,081 at TDFHU and 2,383 at SAPFHU). A representative sample of 363 consultations was calculated for these 6464 WIA, proportionally distributed as 229 for TDFHU and 134 for SAPFHU.

As for the characterization of the users enrolled in these units, there were a total of 17,629 users (9,695 in the TDFHU and 7,934 in the SAPFHU), of which 9,096 (51.6%) were female, 5,011 (51.7%) in the TDFHU and 4,085 (51.5%) in the SAPFHU and 8,533 (48.4%) were male, 4,684 (51.5%) in the TDFHU and 3,849 (48.5%) in the SAPFHU.

In terms of age, most patients were between 18 and 64 years old (10,882 (61.7%)) and were in “active” employment status (7,905 (44.9%)). The remaining distribution of patients in terms of age and employment status is detailed in Table 1.

**Table 1:** Characterization of users enrolled in the units in terms of gender, professional status, and age * Chi-squared distribution; ** Mann–Whitney U test; FHU: Family Health Unit.

Variable		Trilhos Dueça FHU n (%)	Santo André de Poiares FHU n (%)	Total	p
Gender	Male	4,684 (48.3)	3,849 (48.5)	8,533 (48.4)	0.069*
	Female	5,011 (51.7)	4,085 (51.5)	9,096 (51.6)	
Professional status	Active	4,169 (43.0)	3,736 (47.1)	7,905 (44.9)	<0.001**
	Unknown	647 (6.7)	258 (3.2)	905 (5.1)	
	Student	1,279 (13.2)	895 (11.3)	2,174 (12.3)	
	Non-active	1,431 (14.8)	1,074 (13.5)	2,505 (14.2)	
	Non applicable	497 (5.1)	608 (7.7)	1,105 (6.3)	
	Retired	1,672 (17.2)	1,363 (17.2)	3,035 (17.2)	
Age	0-17 years old	1,281 (13.2)	1,050 (13.2)	2,331 (13.2)	0.006**
	18-64 years old	5,882 (60.7)	5,000 (63.0)	10,882 (61.7)	
	≥65 years old	2,532 (26.1)	1,884 (23.8)	4,416 (25.1)	

As for the random and representative sample studied, the average age was 47.0 ± 23.3 years, and the median was 49 years. For the TDFHU, it was 47.6 ± 23.9 years, and the median was 49 years, and for the SAPFHU, it was 46.2 ± 22.7 years and the median was 47 years.

As for the random and representative sample studied, it consisted of n=363 consultations, of which 219 (60.3%) were carried out on female patients, 130 (56.8%) at the TDFHU and 89 (66.4%) at the SAPFHU and 144 (39.7%) on male patients, 99 (43.2%) at the TDFHU and 45 (33.6%) at the SAPFHU.

In terms of age groups, most patients were between 18 and 64 years old (212 (58.4%)) and were working (“active” employment status) (160 (44.1%)). The remaining distribution of users in terms of these two parameters is shown in Table 2.

**Table 2:** Characterization of the sample in terms of gender, age, and type of registration in the units and professional group *Chi-squared distribution; **Mann–Whitney U test; FHU: Family Health Unit.

Variable		Trilhos Dueça FHU n (%)	Santo André de Poiares FHU n (%)	Total	p
Gender	Male	99 (43.2)	45 (33.6)	144 (39.7)	0.070*
	Female	130 (56.8)	89 (66.4)	219 (60.3)	
Age	0-17 years old	40 (17.5)	12 (9.0)	52 (14.3)	0.035**
	18-64 years old	132 (57.6)	80 (59.7)	212 (58.4)	
	≥ 65 years old	57 (24.9)	42 (31.3)	99 (27.3)	
Type of registration	Enrolled	224 (97.8)	131 (97.8)	355 (97.8)	0.972*
	Sporadic	5 (2.2)	3 (2.2)	8 (2.2)	
Professional status	Active	98 (42.8)	62 (46.3)	160 (44.1)	0.347**
	Non active	41 (17.9)	19 (14.2)	60 (16.5)	
	Retired	37 (16.2)	35 (26.1)	72 (19.8)	
	Student	24 (10.5)	7 (5.2)	31 (8.5)	
	Unknown	6 (2.6)	2 (1.5)	8 (2.2)	
	Non applicable	23 (10.0)	9 (6.7)	32 (8.9)	

The average age was 47.5 ± 23.8 years, and the median was 52 years. At the TDFHU, the average age was 45.8 ± 24.2 years, and the median was 52 years. At the SAPFHU, the average age was 50.5 ± 22.9 years, and the median was 54 years.

Almost all the patients were enrolled in the respective units n=355 (97.8%), 224 (97.8%) in the TDFHU and 131 (97.8%) in the SAPFHU.

The sample population was compared with the population enrolled in the units, and a significant difference was found regarding gender (p<0.001), while no significant differences were found regarding profession (p=0.718) and age (p=0.678).

As for the number of acute episodes per patient in this period, most patients used this type of consultation only once, with a maximum of eight uses by one patient (see Table 3).

**Table 3:** Number of consultations per user, by type and in relation to the doctor who gave the consultation (FD or other) *Chi-squared distribution; **Mann–Whitney U test; FHU: Family Health Unit; ADA: Acute Disease Appointment; ISA: Intersubstitution Appointment.

Variable		Trilhos Dueça FHU n (%)	Santo André de Poiares FHU n (%)	Total	p
Number of appointments per patient	1	130 (56.8)	89 (66.4)	219 (60.3)	0.053*
	2	56 (24.5)	28 (20.9)	84 (23.1)	
	3	23 (10.0)	10 (7.5)	33 (9.1)	
	4	12 (5.3)	5 (3.8)	17 (4.7)	
	5	3 (1.3)	1 (0.7)	4 (1.1)	
	6	3 (1.3)	1 (0.7)	4 (1.1)	
	7	1 (0.4)	0 (0.0)	1 (0.3)	
	8	1 (0.4)	0 (0.0)	1 (0.3)	
Type of consultation	ADA	155 (67.7)	55 (41.0)	210 (57.9)	<0.001**
	ISA	74 (32.3)	79 (59.0)	153 (42.1)	
Consultation by the Family Doctor	Yes	108 (47.2)	74 (55.2)	182 (50.1)	0.138**
	No	121 (52.8)	60 (44.8)	181 (49.9)	

**Figure 1:**
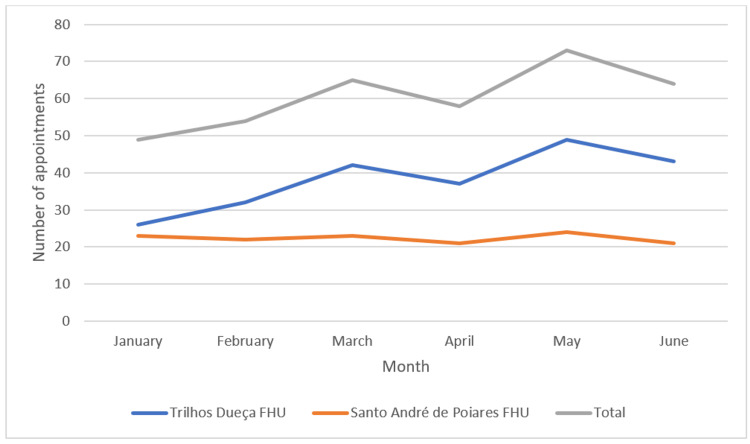
Number of consultations per month p-value: 0.096 (Mann–Whitney U test); FHU: Family Health Unit.

**Figure 2:**
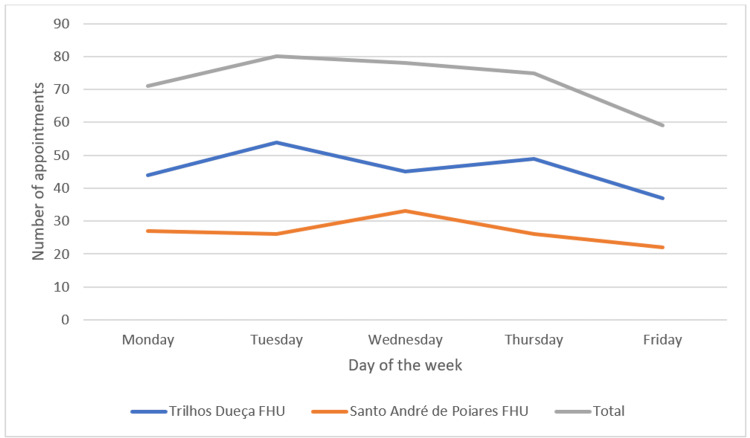
Number of appointments per day of the week p-value: 0.950 (Mann–Whitney U test); FHU: Family Health Unit.

**Figure 3:**
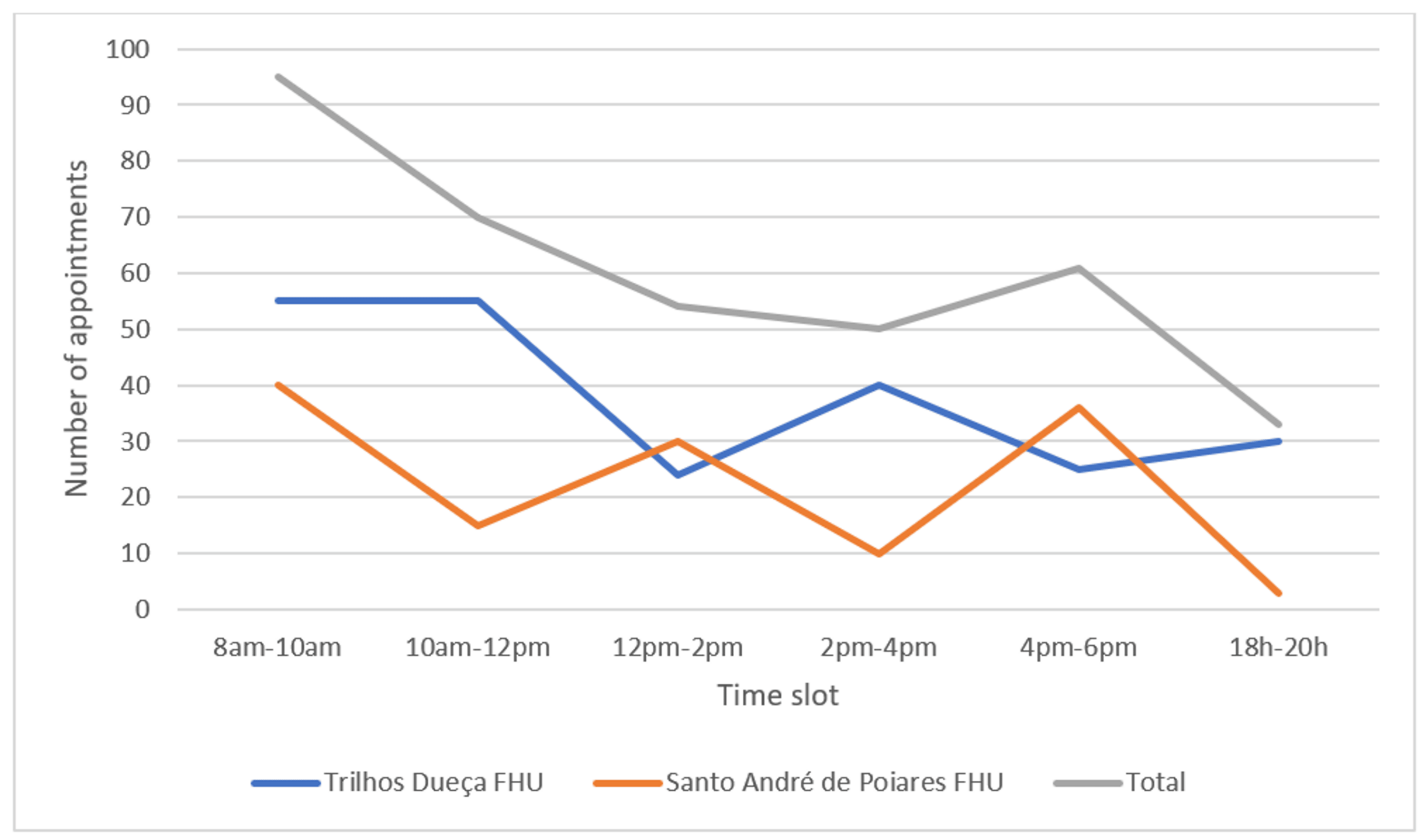
Number of appointments per time slot p-value: 0.635 (Mann–Whitney U test); FHU: Family Health Unit.

In terms of time distribution, May was the month (see Figure 1) with the most WIA, with a total of 73 (20.1%) consultations, and Tuesdays (see Figure 2) were the day of the week with the most consultations, 80 (22.0%). The time slot with the most appointments was between 8 am and 10 am, with 95 (26.1%) appointments (see Figure 3). Most appointments, 182 (50.1%), were carried out by the patient's own FD.

In 140 (38.6%) consultations, the symptoms or their worsening had been present for three days or less and in 105 (28.9%) for more than three days. For the remaining 118 patients (32.5%), 76 (20.9%) did not have the duration of the symptoms described in the records and in 42 (11.6%) of the consultations the reasons were not related to the presentation of symptoms (i.e. they were scheduled to renew sick leave or prescriptions, record the results of complementary diagnostic tests or draw up medical certificates and reports).

Researchers had to classify the reasons for the consultation from the SOAP notes in 303 (83.5%) consultations due to the absence of a recorded classification. The average duration of the symptoms presented was 13.5 ± 39.9 days, with extremes of zero days (symptoms that appeared or worsened on the same day as the consultation) and 365 days, with a median of three days. The mean for TDFHU was 9.4 ± 31.4 days and the mean for SAPFHU was 21.0 ± 51.6 days.

The main reason for consultation by chapter was R “Respiratory” in 109 consultations (30.0%) and by component was R05 “Cough” in 50 consultations (13.8%) (see Table 4).

**Table 4:** Characterization of the five main reasons for consultation and five main diagnoses, duration of symptoms, and referral *Mann–Whitney U test; FHU: Family Health Unit; ER: Emergency Room.

Variable		Trilhos Dueça FHU n (%)	Santo André de Poiares FHU n (%)	Total	p
Five main reasons for consultation (S)		R05 “Cough” 42 (18.3)	L03 “Low back symptom/complaint” 10 (7.5)	R05 50 (13.8)	0.079*
		A04 “Weakness/tiredness general” 22 (9.6)	R05 8 (6.0)	A03 “Fever” 28 (7.7)	
		R21 “Throat symptom/complaint” 19 (8.3)	A03 6 (4.5)	R21 24 (6.6)	
		R07 “Sneezing/nasal congestion” 19 (8.3)	A62 “Administrative Procedure” 6 (4.5)	L02 “Back symptom/complaint” 21 (5.8)	
		U01 “Dysuria/painful urination” 15 (6.6)	A60 “Results Tests/Procedures”6 (4.5)	R07 21 (5.8)	
Duration of symptoms	≤ 3 days	97 (42.4)	43 (32.1)	140 (38.6)	0.071*
	> 3 days	61 (26.6)	44 (32.8)	105 (28.9)	
	Not specified	71 (31.0)	47 (35.1)	118 (32.5)	
Five main diagnoses (A)		A98 “Health maintenance/prevention” 54 (23.6)	A98 22 (16.4)	A98 76 (20.9)	0.042*
		R74 “Upper respiratory infection acute” 33 (14.4)	K86 “Hypertension uncomplicated” 7 (5.2)	R74 37 (10.2)	
		U71 “Cystitis/urinary infection other” 17 (7.4)	P76 “Depressive disorder” 5 (3.7)	U71 20 (5.5)	
		K86 10 (4.4)	L87 “Bursitis/tendinitis/synovitis NOS” 5 (3.7)	K86 17 (4.7)	
		P76 8 (3.5)	R74 /L86 “Back syndrome with radiating pain” 4 (3.0)	P76 13 (3.6)	
Referral (P)	Home	204 (89.1)	114 (85.1)	318 (87.6)	0,233*
	ER	17 (7.4)	12 (8.9)	29 (8.0)	
	Hospital consultation	7 (3.1)	2 (1.5)	9 (2.5)	
	Non-hospital consultation	1 (0.4)	6 (4.5)	7 (1.9)	

By month and component, the most frequent reason for consultation in January was U01 “Dysuria/painful urination” (7.5% of all the coded reason that month); R05 was the most frequent in February (9.0%), March (13.3%), May (13.3%), and June (10.2%); and in April, it was A03 “Fever” (10.1%).

The main diagnostic chapter in this period was A “General and Unspecified” with the components being coded in 96 (26.4%) consultations. By component it was A98 “Health maintenance/prevention” in 76 (20.9%) consultations (see Table 4).

By month, component A98 “Health maintenance/prevention” was the most coded in every month. The second most coded component in January was U71 “Cystitis/urinary infection other” and in the other months, it was R74 “Upper respiratory infection acute”

As for the referral of the patient after the consultation, 318 (87.6%) patients went home (see Table 4). Considering only the WIA deemed appropriate (see the next paragraph), 121 (85.2%) of these patients were sent home.

It was found that 144 (39.7%) consultations did not meet the criteria defined in the Internal Regulations for scheduling WIA (Appendix 1 and Appendix 2). In 142 (39.1%) consultations, these criteria were met and in 77 (21.2%) consultations, it was not possible to characterize this, since the duration of the symptoms was not described in the records of these consultations (see Table 5).

**Table 5:** Appropriate consultation according to the internal regulations *Mann–Whitney U test; FHU: Family Health Unit.

Variable		Trilhos Dueça FHU n (%)	Santo André de Poiares FHU n (%)	Total	p
Appropriate consultation according to the internal regulations	Yes	97 (42.4)	45 (33.6)	142 (39.1)	0.130*
	No	86 (37.5)	58 (43.3)	144 (39.7)	
	Undetermined	46 (20.1)	31 (23.1)	77 (21.2)	

## Discussion

The objectives of this study were to evaluate and characterize the profile of WIA users in the first six months of 2022, to understand the reasons why they went to these consultations, to analyze the diagnosis made, and the referral after the consultation. Unlike other studies carried out on the same subject, it analyzed the adequacy of the reasons for requesting a consultation with the Internal Regulations of each FHU. It was multicentric, two FHUs in the context of a country town, and it was carried out in the aftermath of the COVID-19 pandemic.

According to the results, women were the most frequent users, as in other studies on the same subject [6-11]. People with an “active” professional situation, as seen in other studies [7,10,11] and aged between 18 and 64, were also the main users.

The month of May and Tuesdays were the days with the most appointments, contrary to other studies in which January and Mondays were the busiest [6,7,10]. As in another study from 2021, the first hours of the morning had the highest number of acute illnesses [6]. This was probably because WIA are mostly scheduled for early morning and mid-afternoon, in accordance with the doctors' schedules, and the times of the appointments offered were identical throughout the different days of the week.

In May 2022, at the national level, there was a new peak of new cases of SARS-CoV-2 infection (698,158 cases), after the winter peak. This may justify a greater demand for urgent health care by the population in that month, given that the main reason for consultation in May was R05 “Cough”. Regarding Tuesdays, this is the day with the most WIA in the two FHU, and no justification can be found for this, since it is not the day with the most WIA, nor is it the day of the weekly market in the towns of the two municipalities.

The internal regulations of the two units define the following as criteria for access to WIA (Appendices 1 and 2): people who report the appearance of a sudden/acute symptom that has appeared in the last three days, people who report worsening chronic problems (e.g. decompensation of diabetes mellitus, hypertension, heart failure); trauma, possibly with wounds (need for sutures) and emergency contraception.

According to the results, 102 (28.1%) consultations were for symptoms or worsening of chronic illnesses that had been present for more than three days, and the average duration of the symptoms presented was 13.5 days. For 42 (11.6%) appointments, the reasons were not related to the presentation of symptoms, i.e., they were made to renew sick leave or prescriptions, record the results of complementary diagnostic tests, draw up certificates or medical reports. This means that 144 (39.7%) of the consultations carried out were, according to the internal regulations, inappropriately marked as WIA. One study reported inadequacy rates of 11% [10], and another reported figures of between 18.8% and 46.5% [11]. However, the adequacy criteria of the latter were less strict, with the duration of symptoms considered acceptable at up to 15 days in one and up to four weeks in the other, respectively [10,11].

It will be important to understand the reason for the high proportion of inappropriately used WIA. Some valid reasons are a lack of awareness on the part of users of the reasons for booking this type of appointment, Internal Regulations with excessively tight criteria, and a lack of rigor in booking appointments on the part of clinical secretaries; however, no causal relationships can be established.

It is necessary to raise awareness and educate consultants so that they know the access criteria for each type of consultation. It may also be necessary to revise the FHU's internal regulations, as well as to inform technical assistants of the need to be more rigorous when making appointments. It might also be relevant to implement a screening process to check whether the WIA is appropriate or not, using an instrument to be created and validated.

For 77 (21.2%) consultations, it was not possible to assess whether the criteria for access to an acute illness had been met in relation to the duration of symptoms, as this information was absent from the respective records. In fact, describing the duration of symptoms is crucial not only for assessing the appropriateness of the consultation for its type, but also to enable future research in this field, so it is important to standardize this information in the notes.

It was also found that the main reasons for consultation by chapter were R “Respiratory”, L “Musculoskeletal” and A “General and Unspecified”. By component, R05 “Cough” was the main reason for consultation, followed by A03 “Fever” and R21 “Throat symptom/complaint” in third place. In a 2018 study, it was found that R05 “Cough” was also one of the main reasons for consultation [7]. In addition, R05 “Cough” was the most frequent reason per component in all months, except for January and April.

The three main chapters coded as diagnosis were: A “General and Unspecified”, L “Musculoskeletal” and R “Respiratory”. By component, the most frequent, in descending order, were A98 “Health maintenance/prevention”, R74 “Upper respiratory infection acute”, and U71 “Cystitis/urinary infection other”. A98 is worth mentioning as a diagnosis in a consultation of this type.

By month, the A98 “Health maintenance/prevention” component was the most coded assessment in every month, which implies the importance of discussion and medical training for its implementation as a diagnosis. The second most coded component in January was U71 “Cystitis/urinary infection other”, and in the other months it was R74 “Upper respiratory infection acute”. In fact, although it is not the main diagnosis by component, as is the case in other studies in the area, upper respiratory tract infection is one of the most frequent reasons for using the WIA, the vast majority of which are due to virus infections, which is why patients should be trained to self-manage their pathology, since treatment is essentially symptomatic [6-9]. However, it is assumed that the recent pandemic may have influenced many patients to go to this type of consultation for fear of SARS-CoV-2 infection.

The high coding of A98 “Health maintenance/prevention” as a diagnosis in WIA implies that either patients are going to WIA too often for reasons unsuited to the type of consultation, or programs such as “Oncology Screening” and “Child Health” are opened in WIA, or that the A98 classification is recorded without much discretion on the part of the medical team, due to the obligation to code in A of the SOAP notes, and is a sign of misuse of the classification for reasons of possible work stress.

It is essential that doctors keep good records, both in notes and in ICPC2 classifications. The 15-minute period stipulated for each WIA may be one of the reasons for the absence of important information in S, such as the duration of symptoms and the correct classification of the reasons for the consultation and the diagnoses made. In fact, this period can be short and difficult to manage for the doctor, who ends up unconsciously omitting important information from the consultation, even if he has collected it. It may therefore be necessary to rethink the length of time stipulated for each type of consultation. The importance of standardizing the information to be collected could be a way of reducing this problem by creating a mnemonic for use.

Regarding the referral of the patient after the consultation, 121 (85.2%) of patients using an adequate WIA were sent home, concluding that primary health care can respond to most cases.

Further studies over different time periods and in other regions of the country are necessary, as they will allow us to think about better resource management, adapting the offer of WIA to people's needs, ensuring effectiveness and improving the satisfaction of doctors and people who attend WIA, which takes time away from the proper management of the chronic.

Limitations

The need for the researchers to classify the reasons for consultation, based on the notes in 303 (83.5%) consultations due to the absence of a recorded classification; the ICPC-2 classification is somewhat limited for the exact characterization of some reasons for consultation/pathology, which creates some constraints on the exact perception of the pathology that really motivated the observation; the possible lack of up-to-date data on users' professional status; the lack of duration of the symptoms that motivated the SOAP S observation in 77 (21.2%) of the WIA, making it difficult to assess the suitability of the reason for the consultation to the type of consultation; the assessment of the suitability of the consultations to the WIA type was based on the “a posteriori” records by doctors who were not present at the consultations and not by the doctors who carried them out.

SWOT analysis

Strengths

It stands out from other studies carried out on the same subject because it analyzed the adequacy of the reasons for requesting consultations according to the Internal Regulations of each FHU. It was multicenter, carried out in two FHUs in the context of a country town, and was carried out at the end of the COVID-19 pandemic. Regarding the description of the use of consultations, this is a study that goes into detail to understand the specific reasons for using consultations and then the respective diagnoses. It presents a robust methodology, with a sample calculated for a 95% confidence level, being representative of the universe of consultations. It presents some concrete ideas on how to improve the use of this type of consultation.

Weaknesses

Although the sample was calculated for a 95% confidence level and randomized, a statistically significant difference was found in terms of gender, which may indicate that the sample is not fully representative of the population under study. The need for researchers to classify the reasons for the consultation, based on the notes in 303 (83.5%) of consultations, due to the absence of a recorded classification. The lack of duration of the symptoms that motivated the SOAP S observation in 77 (21.2%) of the WIA, making it difficult to assess the suitability of the reason for the consultation to its typology. Finally, the assessment of the adequacy of the consultations to the WIA typology was based on the “a posteriori” records by doctors who were not present at the consultations and not by the doctors who carried them out.

Opportunities

This study makes it possible to understand the reasons that lead patients to go for WIA and, consequently, the respective diagnoses and whether they fit into the consultation typology, according to the Internal Regulations It was found that 144 (39.7%) of consultations were inappropriately marked as a WIA: for symptoms or worsening of chronic illnesses for more than three days or for reasons unrelated to the presentation of symptoms (renewing sick leave or prescriptions, recording the results of complementary diagnostic tests, drawing up certificates or medical reports). These results are an opportunity to reflect on what is lacking: more health education for users; the need to rethink the criteria for access to WIA in the Regulations; the need to alert clinical secretaries to the importance of rigor in making appointments; the need to implement triage to assess the appropriateness or otherwise of making an appointment. In addition, this study has shown that some important information, such as the duration of symptoms, is being withheld from the S and that there is excessive use of A98 “Health maintenance/prevention” in the A of the SOAP. This is an opportunity to reflect on whether the duration of consultations is adequate and/or whether doctors are suffering from work-related stress that prevents them from making more rigorous records.

Threats

This discusses failure to value and reflect on the results of this study and failure to use concrete ideas for improving the use of acute consultations by other colleagues, coordinators, and even managers at the Local Health Unit level.

## Conclusions

Women used the WIA more often than men did. The main reasons for WIA were related to R “Respiratory”, namely symptom R05 “Cough”. Despite most appointments having been made according to the FHU internal regulations, there are a relative number of appointments not following the criteria defined for attending this type of consultation.

It is necessary and important to have a good and complete electronic record, with standardized notes for the WIA and an appropriate ICPC-2 classification, so that the patient's clinical follow-up is not compromised.

## Appendices

Appendix 1 - Trilhos Dueça FHU Internal Regulations (Walk-in Appointments)

Acute illnesses will be attended to on the same day, within the unit's timetable, preferably by their family team, or in their absence or impediment, by another professional on an inter-substitution basis.

All the doctors and nurses have daily unscheduled consultation periods in their express timetable to attend to these acute cases, and there are also inter-substitution periods at 11 am-12 pm, 1 pm-14 pm, 4 pm-5 pm and 6 pm-8 pm (on Fridays, because there is a service meeting, the mid-afternoon period is from 4.30 pm-5.30 pm). Acute illness consultations last 15 minutes per appointment. In terms of general guidelines, access criteria are considered: (i) People who report the appearance of a sudden/acute symptom that has arisen in the last three days; (ii) People who report a worsening of long-standing problems (e.g. decompensation of diabetes mellitus, hypertension, heart failure); (iii) Trauma, possibly with wounds (need for sutures); (iv) Emergency contraception.

Likewise, the following situations are considered exclusion criteria as they do not fit the criteria for access to this consultation: (i) Showing examinations of any kind, unless related to situations defined in the previous paragraph; (ii) Requesting certificates or declarations, even if urgency is invoked; (iii) Requesting exams/transcription of exams from other doctors; (iv) Requests to renew prescriptions.

If the aforementioned access criteria are met, an appointment will be booked and the user will be told the approximate time of the appointment, calculated according to the clinical urgency and the capacity to respond at the time.

Situations that are considered urgent and/or emergent and are misdirected to the FHU should be prioritized and immediately taken to the observation room and the family doctor and nurse contacted, if the consultation is acute, or the doctor and nurse interchangeably, and then taken to hospital, possibly with the intervention of the INEM (National Institute for Medical Emergencies)."

Appendix 2 - Santo André de Poiares FHU Internal Regulations (Walk-in Appointments)

Appointment for acute illness: (i) unscheduled appointment that can be booked on the same day with the respective family doctor (by telephone or in person), or other doctor (in person) via the internal inter-substitution system in situations where the first option is not possible. All doctors have at least one daily acute illness consultation period, every working day of the week. These periods are duly publicized to patients.

Access criteria: (i) Patients who report a sudden/acute symptom that has appeared in the last three days (fever, cough, vomiting, diarrhea, headache, myalgia, osteoarticular pain, odynophagia, otalgia, lumbago, urinary complaints); (ii) Patients with dyspnea, tachycardia and high blood pressure; (iii) Patients who report a worsening of one of their long-standing problems; (iv) Patients with trauma or wounds without scheduled treatment.

With regard to the need for medical care, the following situations do not fit the criteria for access to this consultation: (i) Transcription of tests ordered by other doctors; (ii) Requests for certificates or declarations of any kind, even if the patient says they are very urgent; (iii) Showing any type of examination or clinical information of hospital or other origin, unless the clinical situation justifies it."
